# Blood-pool MRI assessment of myocardial microvascular reactivity

**DOI:** 10.3389/fcvm.2023.1216587

**Published:** 2023-11-01

**Authors:** Sadi Loai, Beiping Qiang, Michael A. Laflamme, Hai-Ling Margaret Cheng

**Affiliations:** ^1^ Institute of Biomedical Engineering, University of Toronto, Toronto, ON, Canada; ^2^ Translational Biology & Engineering Program, Ted Rogers Centre for Heart Research, Toronto, ON, Canada; ^3^ McEwen Stem Cell Institute, University Health Network, Toronto, ON, Canada; ^4^ Peter Munk Cardiac Centre, University Health Network, Toronto, ON, Canada; ^5^ Laboratory of Medicine and Pathobiology, University of Toronto, Toronto, ON, Canada; ^6^ The Edward S. Rogers Sr. Department of Electrical and Computer Engineering, University of Toronto, Toronto, ON, Canada

**Keywords:** vasomodulation, microvascular function, blood-pool imaging, heart, hypercapnia

## Abstract

**Purpose:**

The ability to non-invasively image myocardial microvascular dilation and constriction is essential to assessing intact function and dysfunction. Yet, conventional measurements based on blood oxygenation are not specific to changes in blood volume. The purpose of this study was to extend to the heart a blood-pool MRI approach for assessing vasomodulation in the presence of blood gas changes and investigate if sex-related differences exist.

**Methods:**

Animals [five male and five female healthy Sprague Dawley rats (200–500 g)] were intubated, ventilated, and cycled through room air (normoxia) and hypercapnia (10% CO_2_) in 10-minute cycles after i.v. injection of blood-pool agent Ablavar (0.3 mmol/kg). Pre-contrast T_1_ maps and T_1_-weighted 3D CINE were acquired on a 3 Tesla preclinical MRI scanner, followed by repeated 3D CINE every 5 min until the end of the gas regime. Invasive laser Doppler flowmetry of myocardial perfusion was performed to corroborate MRI results.

**Results:**

Myocardial microvascular dilation to hypercapnia and constriction to normoxia were readily visualized on T_1_ maps. Over 10 min of hypercapnia, female myocardial T_1_ reduced by 20% (vasodilation), while no significant change was observed in the male myocardium. After return to normoxia, myocardial T_1_ increased (vasoconstriction) in both sexes (18% in females and 16% in males). Laser Doppler perfusion measurements confirmed vasomodulatory responses observed on MRI.

**Conclusion:**

Blood-pool MRI is sensitive and specific to vasomodulation in the myocardial microcirculation. Sex-related differences exist in the healthy myocardium in response to mild hypercapnic stimuli.

## Introduction

The microvasculature plays a vital role in maintaining homeostasis: in addition to being the site of nutrient and gas exchange, it also maintains blood pressure, regulates perfusion in response to locally varying metabolic demands, and ensures new tissue grafts survive *in vivo* (1). In healthy microvessels, vasomodulation is intact, meaning vessels can dilate or constrict as required to increase or decrease perfusion. In many diseases, however, including cardiac and inflammatory conditions, vasomodulatory capacity is impaired, resulting in blunted vasodilation and/or vasoconstriction and ultimately reducing baseline perfusion (2). The current MRI approach for assessing microvascular vasomodulation is to apply a stressor (e.g., acetylcholine or hypercapnia) in tandem with an acquisition sequence sensitive to changes to tissue perfusion (3). Typically, a T_2_*-weighted sequence is applied owing to its sensitivity to the concentration of deoxyhemoglobin (i.e., the BOLD effect), which is taken to be a surrogate index of perfusion. With this approach, cerebral vasoreactivity (4) and coronary vasodilation in the human heart (5) have both been successfully interrogated. Nonetheless, the T_2_*-weighted BOLD signal is non-specific. Changes in local oxygen consumption, blood oxygen saturation, flow velocity, and hematocrit can alter the BOLD effect in the absence of vasomodulation (6).

To specifically measure vasomodulation, one requires a metric that correlates with blood volume but is unaffected by concomitant confounders such as varying local oxygen metabolism. To address this need, Ganesh et al. proposed in 2017 a blood-pool MRI approach for measuring vasomodulation in the presence of changes in blood gases, first demonstrating in abdominal organs (7) and later extending it to ischemic disease (8) and low-perfusion tissue such as skeletal muscle (9). A blood-pool contrast agent is indispensable, as without it, earlier studies have shown low sensitivity and specificity to vasomodulation (10, 11). Using the blood-pool MRI method, one administers a blood-pool contrast agent (e.g., Ablavar) to achieve stable signal in blood vessels and then measure changes in the T_1_-weighted signal or T_1_ relaxation time as the microvasculature dilates or constricts in response to a stimulus. Because Ablavar binds reversibly to serum albumin, the bulk of the gadolinium-based contrast agent is retained in the vasculature, reducing the T_1_ relaxation time of water molecules inside and in close proximity to blood vessels. T_1_ changes are, therefore, a direct result of changes in contrast agent distribution space (i.e., changes in blood volume). Note that with this method, the goal is to obtain relative changes in blood volume in the minutes post-stimulus. The blood-pool MRI approach has seen much traction recently, with reports of its application to swine studies of the heart emerging later, using adenosine as a vasodilator and a different blood-pool contrast agent, Feraheme, to identify coronary perfusion deficit (12).

In this study, we exploit the specificity of the blood-pool approach of (7) to characterize vasomodulation in the healthy heart of male and female rats in response to hypercapnic stimuli. This characterization of the normal heart has not been reported in the literature, and potential sex-related differences are also unknown. Furthermore, to provide evidence that the MRI method is, indeed, tracking vasodilation or constriction, we perform laser Doppler flowmetry, an invasive gold-standard perfusion test.

## Materials and methods

### Animal preparation

This study was approved by the University of Toronto's Animal Care Committee (protocol #20012191). All procedures were conducted in accordance with the Canadian Council on Animal Care. Four- to five-month-old male (*n* = 5) and female (*n* = 5) Sprague Dawley rats (Charles River, Quebec, Canada) were housed three per cage with a 12:12-hour light-dark cycle and constant room temperature (23°C ± 1°C). All rats were fed a standard chow diet *ad libitum* for the duration of the study and had free access to water. Male rats weighed between 400 and 500 g and female rats between 200 and 250 g at the time of imaging. Animals were anesthetized on 5% isoflurane in room air (2 L/min) and then intubated with a 14–16-gauge angio-catheter prior to being transferred to the MRI bed. Respiration was maintained at 80 breaths/minute. Animals were placed in a supine position inside the MRI coil (centered on the heart) and were maintained at 37°C while imaging. Isoflurane was maintained at 1.5%–2% throughout the imaging session. Vital signs (heart rate, blood oxygen saturation, respiration rate) of the animal were monitored in real-time throughout the imaging session using a rodent oximeter (MouseOX Plus, STARR Life Sciences, Oakmont, PA, USA) placed on the hind paw. A 24-gauge tail vein angio-catheter was inserted and secured for contrast agent injection.

### Gas challenge

**Figure 1:**
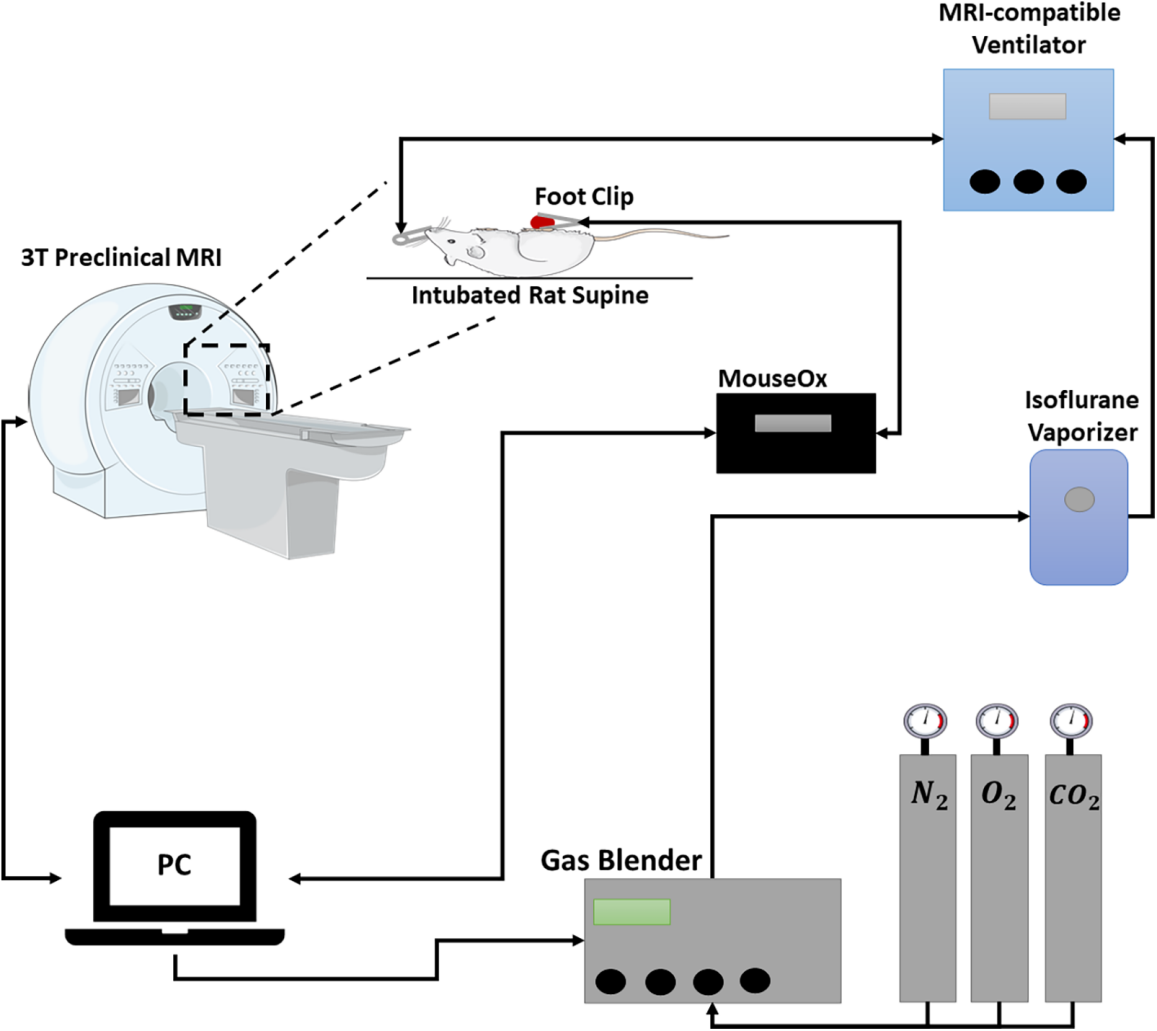
Schematic of gas challenge MRI setup. Gas proportions are controlled by a dedicated computer and gas blender that mixes gases at the desired concentrations and feeds the mixture through an isoflurane vaporizer. The mixed gas is directed towards an intubated rat. A MouseOx is connected to the hind paw to monitor vitals and oxygen saturation. Breathing is controlled through a ventilator.

Elevated carbon dioxide (CO_2_) is a safe and reliable vasodilatory or vasoconstrictive stimulus and can effectively differentiate healthy from diseased microvasculature. Very briefly, 10% CO_2_ was mixed with 21% oxygen (O_2_), balanced with nitrogen in a GSM-3 gas mixer (CWE Inc., Ardmore, PA, USA), and directed to an intubated rat. Respiration was controlled via an MRI-compatible ventilator (MRI-1 Ventilator, CWE, Ardmore, PA, USA); vital signs and blood oxygen saturation were monitored via an oximeter. A schematic of the gas challenge setup is shown in Figure 1. A blood-pool T_1_ contrast agent (Ablavar, Lantheus, North Billerica, MA, USA) was injected intravenously as a bolus (0.3 mmol/kg) followed by a saline flush; this agent eliminates sensitivity to molecular oxygen and produces changes in T_1_ dominated by the blood volume fraction. The extended residency time of Ablavar, which stems from protein binding, allows a prolonged period of stable T_1_ signal enhancement (approximately 40 min) (13, 14).

### In-vivo cardiac MRI

Imaging was performed on a 3 Tesla pre-clinical scanner (MR Solutions, Guildford, United Kingdom). Rats were placed in a designated rat coil, positioned supine and head-first. Localizer scans were first acquired to determine placement of the imaging volume on the cardiothoracic region. The imaging-injection protocol consisted of: (1) baseline 3D T_1_-weighted CINE in the true short-axis plane (FA = 15°, TR = 6 ms, TE = 2.79 ms, 1 average, field of view = 70 mm, 16 slices, slice thickness = 1 mm, matrix size = 128 × 128, pixel spacing = 0.55 mm) and pre-injection T_1_-mapping via a variable flip angle approach (15, 16) (FA = 2°, 5°, 10°, 15°, 20°, TR = 7 ms, TE = 3.36 ms), (2) Ablavar injection followed by a 10 min waiting period for stabilization, and (3) 3D CINE repeated every 5 min. The gas challenge regime post-contrast consisted of 10 min at normoxia (21% O_2_), followed by 10 min at 10% CO_2_, and finally a return to normoxia for 10 min. A specialized cardiac gating software that utilizes solely respiratory motion to gate retrospectively to the rodent's heartbeat was used to reconstruct cardiac images in two phases of the cardiac cycle (17). During reconstruction, a navigator echo incorporated after radiofrequency excitation is used to separate cardiac and respiratory motion. The number of cardiac frames is also set, in this case, to two phases, and analysis was done only on the diastolic phase. A detailed protocol of the cardiac gating software with built-in analysis and open-source code can be found here (18, 19).

### MRI data analysis

Quantitative data analysis was performed in-house using Matlab (v2022, MathWorks, Natick, MA, USA). Post-injection T_1_ relaxation times were computed from pre-injection T_1_ maps and post-injection signal intensity via the spoiled gradient echo signal equation (20). Slice location for image analysis was consistent among all animals. Regions of interest (ROIs) were drawn manually around the septum and the anterior and posterior myocardial wall. T_1_ relaxation times were averaged across all ROIs at individual time-points of the gas challenge protocol.

### Laser Doppler perfusion measurements

In a separate, terminal session, animals underwent real-time perfusion measurements using laser Doppler flowmetry (OxyFlo, Oxford Optronix, Abingdon, United Kingdom) to corroborate MRI results. A novel approach was developed for this technique, as laser Doppler myocardial perfusion measurement had not been attempted previously. A fiber-optic probe (250–450 µm diameter) was secured into the myocardium of the left ventricle; perfusion in arbitrary units was measured dynamically as the rat underwent hypercapnic challenge. The OxyFlo system computes the product of the red blood cell concentration and red blood cell velocity within a small volume around the probe tip, thus yielding a measurement in relative blood perfusion units (BPU). The data was analyzed in LabChart Reader (ADInstruments, Sydney, Australia).

### Statistical analysis

Differences in T_1_ relaxation times throughout the gas regime were analyzed using a one-way analysis of variance (ANOVA) with post-hoc analysis based on the Fisher's Least Significant Difference test. Significance was reported at a *P*-value of 5%.

## Results

**Figure 2:**
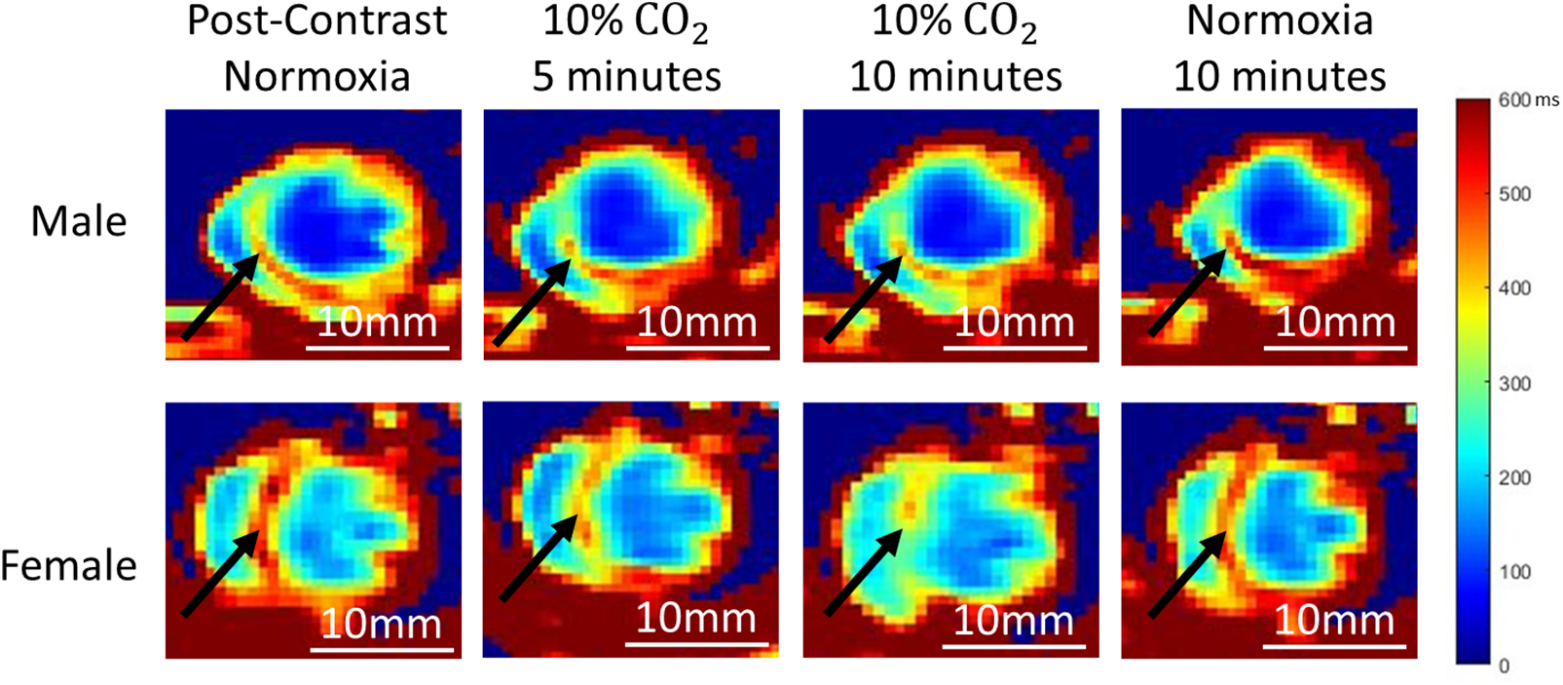
Representative cardiac T_1_ maps at different time-points of normoxia-hypercapnia-normoxia gas stimuli in both sexes. A young (5 months) male (top) and female (bottom) rat are shown. Black arrows highlight signal changes in the septum.

Example T_1_ maps at different timepoints throughout the normoxia-hypercapnia-normoxia gas regime are shown in Figure 2. The septum in both a healthy adult male and female rat visibly demonstrate changes in T_1_ during the transition from hypercapnia (10 min on 10% CO_2_) to normoxia. The T_1_ value increases in both instances (19% ± 5% in males, *P* < 0.05; 23% ± 5% in females, *P* < 0.01), indicating a reduction of microvascular volume occupied by Ablavar (i.e., vasoconstriction). The 10% CO_2_ challenge, however, did not produce consistent responses in the two sexes. While there was a negligible increase in T_1_ in the male septum representative of minor vasoconstriction, the T_1_ decrease observed in the female septum was significant (−20% ± 5%, *P* < 0.05), indicative of an enlarged microvascular volume occupied by Ablavar (i.e., vasodilation).

**Figure 3:**
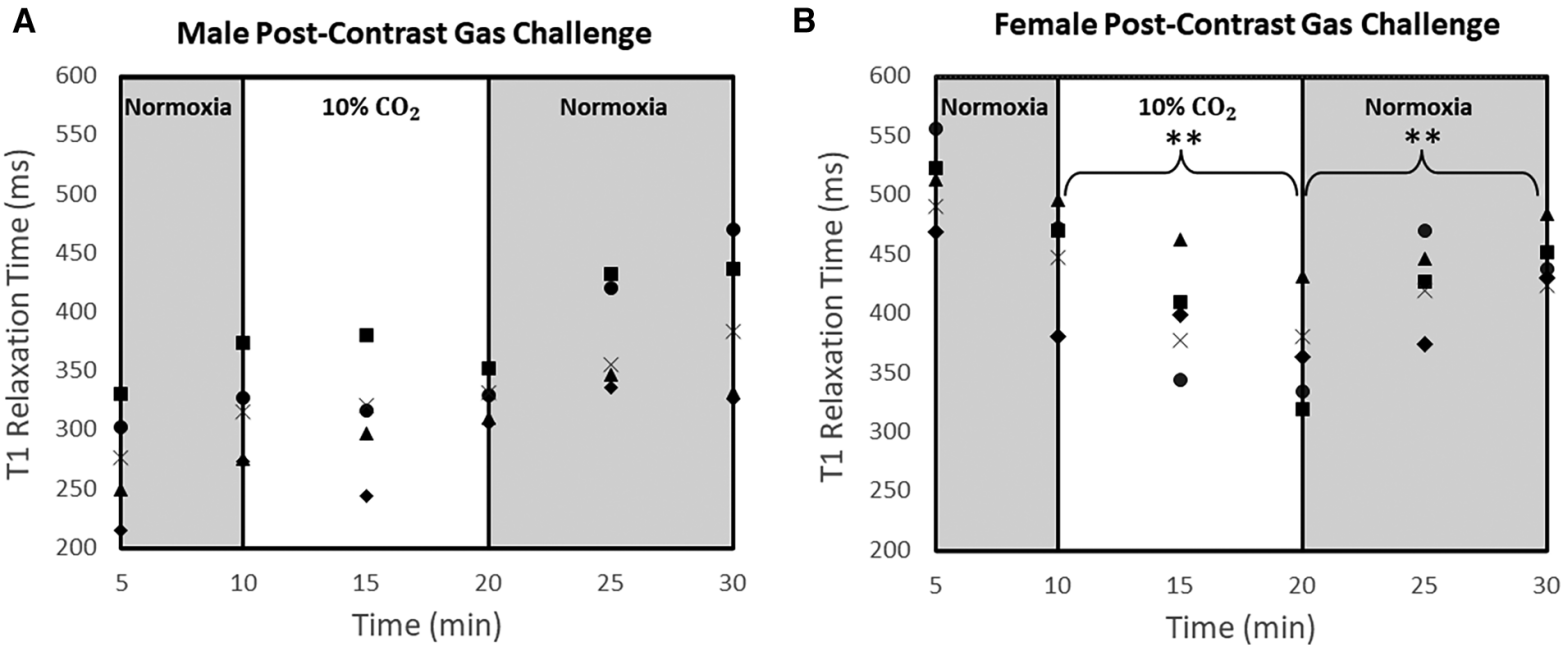
T_1_ measurements at different time-points of normoxia-hypercapnia-normoxia gas stimuli in the myocardium of all rats. T_1_ measurements were averaged over the septum, anterior wall, and posterior wall for each animal at each time point [*n* = 5 male rats (**A**) and *n* = 5 female rats (*n* = 5) (**B**)]. Significance is indicated relative to the start of each 10-minute challenge (**P < 0.01).

T_1_ measurements in individual rats throughout the 30-minute gas regime post-Ablavar are shown in Figure 3. The sex-dependent differences in response to 10% CO_2_ illustrated in Figure 2 can be seen in this larger cohort. Namely, the female rat myocardium demonstrates marked vasodilation, while the male rat myocardium exhibits negligible change. Reversion to room air after hypercapnia elicits vasoconstriction within 10 min in both sexes. Table 1 tabulates the absolute T_1_ relaxation times pre- and post-Ablavar and the percent change in T_1_ as animals transition through different intervals of the gas regime. Notably, the myocardium of female rats exhibited a 20% ± 5% decrease (*P* < 0.05) in T_1_ after 10 min on 10% CO_2_, while that of male rats displayed a 5% ± 3% increase (*P* = 0.2). Upon return to normoxia, a rapid change in T_1_ is observed after 5 min, with an 18% ± 7% increase (*P* < 0.05) in females and 16% ± 4% (*P* = 0.1) increase in males. Oxygen saturation was maintained above 98% in all animals throughout the gas regime.

**Table 1:** Gas challenge and corresponding $T_{1}$ relaxation time measurements (ms).

Gas challenge sequence	Male (*n* = 5)	Female (*n* = 5)
1) Pre-contrast $T_{1}$ relaxation time (ms)	963 ± 55	1,195 ± 28
2) Post-contrast $T_{1}$ relaxation time (ms) - Baseline	312 ± 17**	452 ± 17**
3) % $T_{1}$ change from baseline after 5 min on 10% $CO_{2}$	−1% ± 3%	−12% ± 5%
4) % $T_{1}$ change from baseline after 10 min on 10% $CO_{2}$	5% ± 3%	−20% ± 5%*
5) % $T_{1}$ change from hypercapnia after 5 min on normoxia	16% ± 4%	18% ± 7%*
6) % $T_{1}$ change from hypercapnia after 10 min on normoxia	19% ± 5%*	23% ± 5%**

Percent $T_{1}$ changes are calculated relative to the previous imaging timepoint, with significance indicated (*P < 0.05, **P < 0.01) relative to the previous gas challenge. Data represented as mean ± SEM.

**Figure 4:**
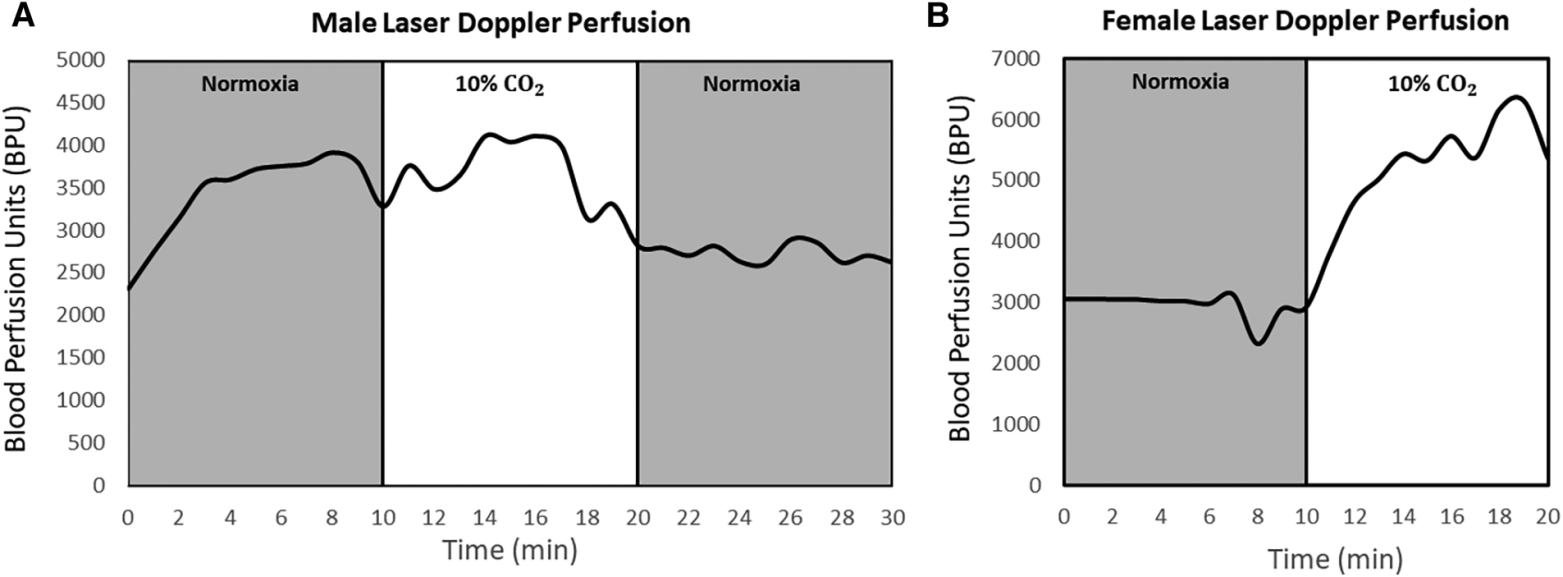
Laser Doppler perfusion measured continuously during normoxia-hypercapnia-normoxia gas stimuli in the myocardium in both sexes. Data for a representative male (**A**) and female (**B**) rat are shown. Measurements were collected at one reference location in the left ventricle for each animal at a frequency of 10 Hz. The transition from hypercapnia to normoxia in female rats was omitted due to noise and data corruption from extensive heart movement. Units are in relative blood perfusion units (BPU).

Percent changes in BPU and heart rate as animals transitioned through gas regimes are tabulated in Tables 2,3, respectively. Upon transitioning to 10% CO_2_ for 10 min from baseline, female myocardium experienced a 33% ± 17% increase (*P* = 0.2) in perfusion, while male rats experienced a 12% ± 16% decrease (*P* = 0.6) in myocardial perfusion. After transitioning back to normoxia for 10 min, male myocardium saw an additional 24% ± 11% reduction (*P* = 0.6) in perfusion, while perfusion measurements were corrupted in females due to significant motion of the heart against the optical probe. Real-time laser Doppler perfusion measurements from representative rats are shown in Figure 4. Percent change in female heart rate was minimal throughout the experiment, with average percent change remaining under 5% (*P* = 0.3). Male heart rate, on the other hand, increased 11% (*P* = 0.2) during the 10-minute 10% CO_2_ challenge.

**Table 2:** Gas challenge and corresponding change in laser Doppler perfusion measurements.

Gas challenge sequence	Male (*n* = 3)	Female (*n* = 3)
1) % change from baseline after 10 min on 10% $CO_{2}$	−12% ± 16%	33% ± 17%
2) % change from hypercapnia after 10 min on normoxia	−24% ± 11%	not measured

Percent changes in perfusion are calculated relative to the previous imaging timepoint. ta represented as mean ± SEM. Measurements in female myocardium during the second challenge were unreliable due to significant movement of the heart against the optical probe in all female rats.

**Table 3:** Gas challenge and corresponding change in heart rate measurements.

Gas challenge sequence	Male (*n* = 3)	Female (*n* = 3)
1) % change from baseline after 10 min on 10% $CO_{2}$	11% ± 6%	4% ± 2%
2) % change from hypercapnia after 10 min on normoxia	1% ± 2%	5% ± 2%

Percent changes in heart rate are calculated relative to the previous imaging timepoint. Data represented as mean ± SEM.

## Discussion

The use of a blood-pool contrast agent to assess microvascular blood volume is not a new concept; in fact, its distinct value was best demonstrated in pathologies where vessels were structurally compromised, as in cerebral neoplasms (21). However, the concept of blood-pool imaging to image vasomodulation in the presence of changing blood gases (e.g., hypercapnia or breathing maneuvers) is relatively new (7–9). Our study provides the first attempt to confirm this technique in the heart using a preclinical gold-standard assessment of perfusion, namely, laser Doppler flowmetry. We demonstrate that our blood-pool method can detect both vasodilation and vasoconstriction, with T_1_ changes on MRI corroborated by changes on laser Doppler perfusion. Furthermore, while the sensitivity of the blood-pool imaging technique was recently demonstrated in the diseased myocardium in response to adenosine (12), the present study shows that hypercapnia-induced vasoreactivity is also sensitively detected using this method, in the absence of disease. Gas challenge has the added benefit of enabling bi-directional blood volume changes, a key feature that is impossible with adenosine stress testing.

Another notable finding is the presence of a sex-dependent difference in the myocardial microvascular response to hypercapnia, specifically, mild hypercapnia (10% CO_2_). In healthy adult rats, the myocardium exhibited significant vasodilation in females but showed no changes in males. Vasodilation at this hypercapnic level is expected in the heart, because in contrast to other organs where 10% CO_2_ induces vasoconstriction, the heart possesses protective properties against hypercapnic acidosis (22). This response, known as “vascular steal,” is critical to facilitating blood flow redistribution and maintaining venous return. In fact, clinical studies have demonstrated increased coronary blood flow and vasodilation is seen at inspired CO_2_ levels as high as 12% (23), a phenomenon attributed to nitric oxide-mediated peri-vascular smooth muscle relaxation (24). On the other hand, blunted vasodilation in the male myocardium was unexpected and contradicts current clinical and pre-clinical studies that claim hypercapnia leads to an increase in myocardial perfusion in males. One possible explanation for this discrepancy could be that because the female heart, unlike the male heart, has a smaller cardiac output and a smaller microvascular blood reserve (25, 26), vasodilation is required to meet metabolic demands, whereas the male heart can better tolerate the added stress. Another explanation is that conventional methods for assessing vasomodulation (i.e., BOLD MRI) measures blood oxygenation and not blood volume. It is possible an increase in BOLD signal stems purely from elevated blood oxygenation, which is a known phenomenon during hypercapnia due to a reduction in heart rate and blood pressure (27, 28).

Laser Doppler perfusion measurements confirmed the specificity of the blood-pool MRI method to vasomodulation. It is important to note that while perfusion is not equivalent to blood volume, changes in perfusion normally follow changes in blood volume in the absence of drastic shifts in flow velocity. For example, in response to 10% CO_2_, female myocardial T_1_ decreased 20% (vasodilation) and male myocardial T_1_ increased 5% (vasoconstriction). These two divergent responses were confirmed on laser Doppler, which revealed 33% increased perfusion in females and 12% decrease in perfusion in males. The MRI and laser Doppler measurements do not correspond one-to-one, because while MRI is sensitive to blood volume, laser Doppler is sensitive to blood volume and blood velocity. Therefore, changes in heart rate, as seen in a 11% elevation in males to 10% CO2, may affect laser Doppler measurements. Upon return to normoxia following hypercapnic challenge, the 19% increase in T_1_ in the male heart corresponded with a 24% decrease in perfusion. However, in the female heart, laser Doppler perfusion measurements were unreliable and could not be used to corroborate the significant vasoconstriction seen on MRI. Errors are incurred on laser Doppler flowmetry when there is non-negligible motion between the heart and optical probe. In the case of the female heart, transitioning from CO_2_ to normoxia introduced irregular cardiac motion that compromised laser Doppler readings and degraded them to the noise level. Despite this sensitivity to erratic motion, our unique surgical design generally enabled stable optical measurements on a beating heart, the first report of its kind.

There are several other noteworthy comments. First, vasomodulatory response to hypercapnia is relatively slow in the heart (over minutes) compared to that in the brain (over seconds), which necessitates the stimulus to be sustained long enough to be effective. Second, female rats had a considerably higher baseline T_1_ compared to males, which can be attributed to a higher collagen content in young, healthy rodent female hearts (29), along with higher extracellular volume, larger gaps in between cardiomyocytes, and significantly lower hematocrit levels when compared to their male counterparts (30). Third, the lack of vasodilation in response to mild hypercapnia in males may not persist throughout the lifespan: it is entirely possible the response may change to resemble that in females as male rats age. This highlights the importance of understanding what defines normal vasomodulation in different age groups. Fourth, Ablavar is a reversible albumin-binding agent, which means it is not purely intravascular. When it transiently binds albumin and then unbinds, Ablavar will cross the vascular endothelium and occupy a space greater than the blood volume. While reversible albumin-binding precludes absolute quantification of blood volume, it enhances detection sensitivity by allowing a greater pool of water molecules to access the contrast agent. In the presence of vessel leakage due to endothelial dysfunction, a larger blood-pool nanoparticle may be a better agent for probing vasoreactivity, while an albumin-binding blood-pool agent can be retained for assessing endothelial leakage. Lastly, we want to emphasize that when analyzing trends in T_1_ or T_1_-weighted signal, it is important to focus on the changes, and not absolute T_1_ times, within minutes post-hypercapnic stimulus, as T_1_ will be subject to error from background drift and contrast elimination over tens of minutes. Previous studies confirmed that within a window of 40 min, T_1_ changes in the intravascular space from contrast elimination were not significant (7).

### Limitations and future work

The procedures involved in this study – intubation, thoracic surgery for laser Doppler flowmetry – were very demanding, and complications often arose that reduced the number in our cohort ultimately to five male and five female rats for imaging, and three rats of each sex for optical validation. Furthermore, our animals were imaged at one timepoint only due to the terminal nature of the perfusion study, which had not previously been attempted for cardiac perfusion in a rat model. Standard deviations were substantial due to the rapid motion of the rodent heart (>300 bpm). Future studies will include time-course imaging in a larger cohort of animals to assess potential changes in myocardial vasomodulation as a function of age. A disease model will also be included to assess the sensitivity of the technology in distinguishing microvascular dysfunction from normal vasomodulation. Translation to the clinic will not require intubation or invasive interventions, as gas challenge intervention is clinically approved and routinely studied (31). Lastly, as the contrast agent used in this study was recently discontinued by the manufacturer and is not available for clinical use, future clinical studies will require an approved blood-pool MRI contrast agent.

## Conclusion

This proof-of-concept study described a novel blood-pool MRI approach for specific assessment of microvascular vasomodulation in the myocardium in the presence of evolving blood gases. Results in young, healthy male and female rats demonstrated our approach was sensitive and specific to vasodilation in response to mild hypercapnia in females, and to vasoconstriction upon return to room air in both sexes. This non-invasive technology holds promise for assessing microvascular dysfunction in different cardiac conditions.

## Data Availability

The raw data supporting the conclusions of this article will be made available by the authors, without undue reservation.
